# The Effect of a Knowledge-Based Intervention on the Use of Respirators in the Norwegian Smelter Industry

**DOI:** 10.3389/fpsyg.2020.00270

**Published:** 2020-02-20

**Authors:** Øystein Robertsen, Marit Nøst Hegseth, Solveig Føreland, Frank Siebler, Martin Eisemann, Hans Christian Bones Vangberg

**Affiliations:** ^1^Department of Occupational and Environmental Medicine, University Hospital of North Norway, Tromsø, Norway; ^2^Department of Psychology, Faculty of Health Sciences, UiT The Arctic University of Norway, Tromsø, Norway

**Keywords:** intervention, respiratory protective equipment, theory of planned behavior, industry, smelting

## Abstract

**Introduction:**

The present study investigated the effect of interventions aiming to improve attitudes toward the use of respiratory protective equipment (RPE), knowledge of RPE and the use of RPE in the Norwegian smelter industry.

**Method:**

The surveys received 567 respondents to baseline and 240 respondents 2 weeks after the intervention. Participants were invited to either a fit-testing of respirators [Group 1] or a fit-testing combined with a lecture on exposure [Group 2], health effects and RPE. The control group [Group 3] received no training. Questionnaires containing measures of subjective knowledge, attitudes and behavior regarding RPE use were assessed.

**Results:**

Testing indicated an improvement in knowledge of RPE and a reduction in perceived inconveniences regarding the use of RPE for both intervention groups. Group 1 showed an improvement in attitudes and organizational support, while intervention Group 2 showed an improvement in subjective norms related to RPE use. Intention to use or rate of respirator use was not shown to change significantly for any group using paired testing. Regression analysis indicated that participation in either intervention influenced intention to use respirators. The effect was significant for Group 1 and was marginally significant for intervention Group 2.

**Conclusion:**

The results indicate that interventions can increase workers’ knowledge and attitudes, and reduce perceived inconvenience regarding the use of respiratory protective equipment. However, even though some variables seemed to positively change, reported respirator use did not improve for either groups participating in the study. It may be that physical barriers with regards to using RPE, such as fogging of protective goggles, sweating, breathing and communication issues outweigh individual attitudes, intentions and social pressure to use respirators.

**Practical Applications:**

The tailored course and practical training in RPE use in the current intervention can be applied in the smelting industry to provide up to date information on dust exposure, health effects and protective equipment. Some adjustments may be warranted for the content to fit specific risks and exposures of other industries. However, the general pedagogical framework of the educational material regarding health effects and RPE should be useful for most heavy industries.

## Introduction

The presence of respiratory risk factors such as gases, fumes, fibers and dust, including nano-sized particles, in the work atmosphere of smelters in the Norwegian metal alloy industry has been well documented (Ellingsen et al., 2003; Føreland et al., 2008; Berlinger et al., 2015; Kero and Jørgensen, 2016; Kero et al., 2017). Depending on the end product and production processes, these exposure factors vary both qualitatively and quantitatively between smelters, but the respiratory risk they represent is of general concern for the industry. Previous studies conclude that Norwegian smelter workers are more susceptible to develop respiratory diseases such as chronic obstructive pulmonary disorder (COPD) than the general population (Johnsen et al., 2008; Søyseth et al., 2011, 2013, 2015). Although measures have been taken to reduce exposure, there are still areas and work-tasks where exposure is too high and occupational exposure levels are exceeded. Indeed, 98% of the employees at Norwegian smelters reported that they were exposed to one or more respiratory health risk factors more than once a week (Hegseth et al., 2018). According to the hierarchy of controls, the use of personal protective equipment (PPE) should be the final solution to end respiratory challenges. Prior to the use of PPE, removal or substitution of hazardous materials, engineering controls or administrative controls should be considered. Some exposure cannot be controlled by engineering or administrative solutions, making personal protective equipment a necessity. All Norwegian smelters have included the use of respiratory protective equipment (RPE) in their health and safety regulations. Yet, compliance with RPE regulations is sub-optimal. Seventy-eight percent of the workers report that they do not always use RPE in exposed situations (Hegseth et al., 2018). Sub-optimal RPE use has been reported in other professions such as nursing, farming, mining, construction and nuclear energy (Salazar et al., 2001; Carpenter et al., 2002; Bryce et al., 2008; MacFarlane et al., 2008; Mitchell and Schenker, 2008; Tam and Fung, 2008; Han and Kang, 2009; Guseva Canu et al., 2013).

In a review by Graveling et al. (2011), the authors suggested several measures to optimize RPE use and compliance among workers. The role of management with regards to facilitating use, providing the correct equipment and training in addition to aspects of the RPE equipment such as user comfort and technical appropriateness were pointed out. Also, correct RPE use could be facilitated through increasing employee intentions by means of education in addition to administrative measures (Szeinuk et al., 2000). Testing for proper RPE fit is important. Facial structures vary individually, and RPE that fits one person adequately may not do so on another individual. The Health and Safety Executive (HSE) standard for fit-testing recommends quantitative fit-testing and suggests a fit-factor of 100 in order to ensure proper protection. Fit-factor is the ratio between ambient particle count and particle count measured inside RPE. Furthermore, RPE fit testing should be performed yearly in order to ensure protection, i.e., if a person gains or lose weight it may affect fit (HSE, 2012).

According to the theory of planned behavior (TPB) (Ajzen, 1985, 1991), behavior is guided by intention, influenced by the antecedents attitudes toward the behavior, subjective norms and perceived control. Attitudes toward behavior emerge from the affective or cognitive evaluations a subject makes regarding the behavior, e.g., feeling comfortable, regarding it as meaningful. Attitudes also involve evaluations of outcomes of performing behavior. Subjective norms are individual evaluations of how the social norms regarding the behavior, this concept comprise descriptive and injunctive norms. For instance, descriptive norms are individual perceptions of how colleagues behave, while injunctive norms are individual perceptions of what colleagues think about a behavior, i.e., whether colleagues approve or disapprove of the behavior. Perceived control is the extent to which the subject feels in control of the behavior, e.g., belief that they can successfully perform the behavior or whether they feel that they control behavior, or if it is externally occurring. These three structures are related to background factors such as personality, intelligence, values, education, culture etc. Therefore, a change in these factors should influence a behavioral change. The TPB has been shown to be effective in predicting behavior and intentions based on attitudes, subjective norms and perceived behavioral control (Godin and Kok, 1996; Armitage and Conner, 2001). Indeed, interventions based on TPB have previously shown efficacy in increasing intentions with regards to health-related behaviors (Brubaker and Fowler, 1990; Murphy and Brubaker, 1990; Fishbein et al., 1996; Jemmott et al., 1998).

Interventions containing training and/or education to influence the use of RPE have previously been performed in farming and health care (Gjerde et al., 1991; Carrico et al., 2007; Dressel et al., 2007; Donham et al., 2011; Kim et al., 2012; Shamsi et al., 2015). A review by Luong Thanh et al. (2016) revealed that given the current knowledge status, there was not sufficient evidence to conclude that training and education interventions *did not* have an effect on the use of respirators. The authors also requested more rigorous studies on this topic. Lunt et al. (2011) came to a similar conclusion when reviewing studies using interventions to improve behaviors related to dermal and respiratory hazards. Mullan et al. (2015) reviewed studies investigating the efficacy of various theory-based interventions in the construction business and concluded that interventions employing feedback, monitoring and goal setting were more effective than behavior instruction and information regarding health effects.

Fit-testing respirators in order to assure sufficient protection has been conducted for some time. As previously mentioned, objective fit-testing is done by using instruments that count particle ratios between the atmosphere inside the respirator and outside. There is evidence that RPE efficiency can be increased by fit-testing respirators on end users (Myers et al., 1995; Or et al., 2012; Harber et al., 2013). The purpose of fit-testing is to prevent leakage of hazardous substances into the RPE (HSE, 2012). Fit-testing is normally done in a one-to-one scenario where the participant receives personalized advice and equipment testing (HSE, 2012), interactions where subjects receive information and testing by a professional can to some extent function as tailored interventions. Tailored interventions demonstrably exert a significant impact on health behavior (Noar et al., 2007). Experience from our clinic suggests that fit-testing increases awareness of RPE and its use. This observation supports the hypothesis that fit-testing may function as an intervention to increase positive behavior. Additionally, as Howie (2005) pointed out, informing the workers of the consequence of exposure is of great importance in increasing motivation to use RPE. Hence, education on relevant exposure and potential health effects could improve the effect of the intervention (Szeinuk et al., 2000).

The overall goal of this study was to improve the rate of RPE use in the Norwegian smelter industry through a tailored knowledge-based intervention comprising a seminar on dust exposure and health effects and/or RPE fit-testing. The aim of this research was to determine which of the described interventions, if any, increased the rate of RPE use.

## Materials and Methods

### Participants

Participants were recruited from the population of smelter workers involved in the DeMaskUs project (Sintef, 2015). Criteria for participation was age of 18 or above and that they had previously worked in or currently were working in jobs where they could encounter respiratory exposure. See Tables 1 and 2 for demographics and plant distribution.

**TABLE 1:** Number of participants and response rate (%) by plant and intervention group at post-intervention.

	Plant 1	Plant 2	Plant 3	Plant 4	Plant 5	Plant 6	Total
Invited	79	58	110	114	228	112	701
Response	33/42%	34/59%	51/46%	43/42%	65/29%	11/10%	237/35%*

	**Control**		**Intervention 1**		**Intervention 2**		

Invited	220		240		241		701
Percent of total	31.38%		34.24%		34.38%		100%
Response	76/35%		69/29%		83/35%		228/33%**

*Not included in the table were 3 wrong codes where participants failed to use the correct code for participating plants. They were however included in the analysis, as they correctly specified which group they belonged to. **Nine participants reported to not having received respirator fit testing in the current study, but having received the lecture intervention and were therefore not included in the analysis.

**TABLE 2:** Sample demographics, by group for participants who responded to both pre and post-intervention questionnaire.

Variable			T1		
			
	*n*		C	I1	I2
Sex	146	Male	49/85.19%	43/89.58%	48/90.38%
		Female	9/14.81%	5/10.42%	5/9.62%
Age	154	(n)/mean/SD	58/44.67/12.19	48/43.38/12.63	53/46.15/14.01
Relationship status	152	Single	14/24.53%	10/21.28%	10/19.23%
		Married	28/49.06%	21/44.68%	31/59.62%
		Cohabitant	12/20.75%	15/31.91%	11/21.15%
		Separated/Divorced	3/5.66%	1/2.13%	1/2.13%
Employment status	155	Permanent employee	56/94.55%	44/91.67%	46/86.54%
		Substitute/Temp	1/1.82%		1/1.92%
		Apprentice	2/3.64%	4/8.33%	6/11.54%
Number of children	151	0	15/26.42%	15/31.25%	17/32.00%
		1	12/18.87%	9/18.75%	5/10.00%
		2	17/32.08%	13/27.08%	16/32.00%
		3	10/19.98%	11/22.92%	12/24.00%
		More than 3	4/5.66%		1/2.00%
Education	155				
		Primary school	3/5.45%	4/8.33%	5/9.62%
		High-school/Vocational school	31/52.73%	420/67%	26/48.08%
		Vocational diploma	20/32.73%	11/22.92%	12/23.08%
		University 3 year	2/3.64%	10/20.83%	6/11.54%
		University 5 year	3/5.45%	3/6.25%	4/7.69%
Number of years experience	155	(n)/Mean/SD	(55)/20.76/2.78	(48)/20.02/12.72	(52)/23.87/13.36

### Design

The presented work was a controlled before-and-after study included in the DeMaskUs project, conducted in the Norwegian smelter industry. The current study described the relationship between intervention participation and intention to use RPE 2 weeks after the intervention, controlling for TPB factors. Furthermore, participants scores on single-item factors were investigated to see if they had changed between baseline and post-intervention.

### Randomization

Participants were randomly assigned into three groups, one control group and two intervention groups, using a random number generator. An intra-plant design was adopted to mitigate influence from confounding variables present at the plants. That is, participants were randomized into three groups at each plant. Interventions were implemented at the six participating plants following the baseline data collection (Robertsen et al., 2018). Smelting plants are operational 24 h, 365 days a year, meaning most participants had shift-arrangements working morning, afternoon, night-shifts or off-days on rotation. The period for data collection and intervention implementation had to be scheduled with the plant management to be conducted at an appropriate time. This meant that during intervention, some shifts had off-days. A completely random allocation of all participants at each plant was therefore not practically obtainable. However, as participants were not deliberately assigned to shifts ahead of time, the randomization was as rigorous as possible.

### Questionnaires

Questionnaires were used to collect data from participants before and after the intervention. Baseline data had been collected using a questionnaire described in Robertsen et al. (2018), comprising scales measuring TPB variables, safety climate, work experience and items measuring perceived exposure, organizational perception regarding support and communication, knowledge and perceived inconveniences regarding the use of RPE. Follow-up data formed the basis for the current article and was collected 2 weeks post intervention. The follow-up questionnaire was a condensed version of the baseline questionnaire comprised of variables thought to change due to the intervention. Primarily TPB factors and single-item factors. Additional items asking if participants had received fit-testing and/or course were added to determine which group they had been assigned to.

#### Factors Based on the Theory of Planned Behavior

Questionnaire items representing the TPB (Theory of Planned Behavior) factors (attitudes toward the use of respirators, subjective norms regarding the use of respirators, perceived control in regards of respirator use, intention to use respirators and rate of respirator use) had been developed in several steps following recommendations by Fishbein and Ajzen (2009); see Robertsen et al. (2018) for details. The factors were employed in the follow-up questionnaires to investigate changes between pretest and posttest as a result of the intervention. Factor sample items; Attitudes (e.g., Regularly using a respirator during the next work-week would be. Very harmful vs. Not harmful at all), Subjective norms (e.g., My colleagues always wear respirators during the work-week.), Perceived control (e.g., It’s up to me whether or not I use the respirator during a work-week.), Intention (e.g., I am going to use the respirator in all required situations next work-week).

See Supplementary Appendix Table A2 for a list of TPB factors and items.

#### Factors Based on Single Items

The single item creation was described in Robertsen et al. (2018). Items aimed at measuring change due to intervention were included in the follow-up. Items were assigned into three factors, see Supplementary Appendix Table A1. Items were scored on a seven point likert-scale from 1,*“Completely disagree”* to 7, *“Completely agree.”*

##### Knowledge

Items in this factor measured perceived knowledge regarding the use of respirators. High scores were favorable and indicated that participants perceived to know more about respirators. Example items; “*I know what the respirator protects against*,” “*I am confident that the respirator works as intended*.”

##### Organization

This factor provided a measure of how participants experienced organizational climate regarding health and safety at work. High scores on these items were considered positive. Example items: “*The organization is willing to provide personalized protective equipment*,” “*Employee suggestions on improvements are taken into consideration properly and discussed openly*.”

##### Inconveniences

The factor measured practical issues regarding the use of respirators, e.g., impractical in certain work-tasks, communication issues or accessibility. Items were negatively worded. A low score indicated that the participants felt less practical obstruction regarding RPE use. Example items: “*It is impossible to follow respirator guidelines during some work-task*,” “*Sometimes I don’t bother changing respirator even though I know I should*.”

The single item factors were thought to be linked to the use of RPE. Knowledge of RPE and perceived organizational support were considered important background factors facilitating use of RPE and perceived inconveniences was thought to directly influence RPE use. A confirmatory factor analysis was performed to check for convergent and discriminant validity of the three factors based upon single-items. Self-reported measures were chosen due to potential problems with biases and cost-effectiveness, monetary and timewise.

### Data Collection and Preparation

Questionnaires were individually packaged and distributed to the plants by the researchers. Contact personnel at each plant distributed questionnaires to the employees. Envelopes contained questionnaires, information leaflet describing the project, what participation entailed and information about chances to win lottery prizes. Baseline questionnaires were sent out 3 weeks prior to the intervention and handed in before the intervention started. Follow-up questionnaires were sent out 2 weeks after the intervention and the participants had 10 days respond.

Returned questionnaires were optically read at the University Hospital of Northern Norway’s Clinical Research Department.

To increase response rates, participants had a chance to win a gift certificate (6 per plant) of approximately 80 EUR.

### Procedures

Participants who had received fit testing prior to the DeMaskUs Project were excluded from the analysis (*n* = 11). In order to follow participants from baseline to follow-up but still keep their anonymity, they were asked to use a self-generated ID-key (Yurek et al., 2008). Participants were invited to take part in the intervention groups, there was no mandatory or forced participation.

### Content of Intervention

#### Intervention 1 (Group 1)

Fit testing was performed using a TSI Portacount Respirator Fit Tester 8038 (TSI inc, Shoreview, MN, United States). The Portacount generates a ratio between measured ambient particle count and particle count inside the RPE, while the wearer executes a standardized set of exercises (HSE, 2012).

Participants were invited to a testing room where the HSE fit test standard (HSE, 2012) was explained to the participants. They were asked which respirator they normally used, if they liked it and how they perceived performance, and they were asked to don the respirator.

The participants could follow the results on screen and for each task the Portacount calculated the fit-factor and indicated either fail or pass. This allowed participants to observe how different tasks affected the fit of the respirator, and how the respirator performed.

If the respirator failed fit-testing, the participant could test a number of other respirators. If the respirator passed testing, we asked if they wanted to test any other respirators to see if they liked them better, and if so another test was performed. The tests were performed by the research group, all of whom had training in the use of the Portacount Pro + machine and HSE-standard. Personnel performing the tests also provided information about how the respirators function, different types of filters and other relevant information about respirators during the test session.

#### Intervention 2 (Group 2)

Group 2 received the same fit-testing procedure as group 1 and in addition they were invited to take part in a 45 min lecture on exposure and health effects named “Dust and Health,” tailored to the production and specific exposure risks at their smelting plant. The course included a brief introduction to the following topics:

• What is dust, fumes and gasses? Specific focus on nanosized particles.
• Where and what kind of dust/fumes/gases were located in the plant.
• How exposure affects the respiratory system.
• Mechanisms behind the development of COPD.
• Description of other health risks related to dust/fumes/gases.
• The meaning of increased risk.
• References to recent studies from their industry.
• Measures to reduce exposure.
• Respiratory protective equipment: Design and structure.
• Properties of different types of filters and RPE.
• The importance of fit-testing.
• Visualization of the amount of nano-sized particles that one can breathe in, by showing a used filter from a dust measurement in an actual smelter.

A toxicologist and a specialist physician in work and occupational medicine delivered the course to the participants. Questions were welcomed both during and after the course. Meeting rooms were booked on site at each participating plant for the delivery of the course and fit-testing. Group 1 were invited to receive fit-testing a specific time slots each day the project personnel were present at the site. Participants in Group 2 were invited to participate in the course prior to being scheduled for fit-testing. Approximately 6–10 participants were invited to each course.

### Control

The control group was not invited to any activity conducted by the researchers or the project. To our knowledge, none of the participating plants conducted respirator fit-testing during the project period.

### Statistical Analyses

Eleven participants stated that they had previously received fit-testing prior to the current study and were excluded. Demographic distributions between groups over time were investigated with Case-control studies odds ratios with Fisher’s exact *p*.

Confirmatory factor analysis was used to determine psychometric properties of the proposed factors containing single items. Raykov’s Reliability Coefficient was applied to investigate convergent reliability, values should exceed 0.70 (Mehmetoglu and Jakobsen, 2017, p. 304). Furthermore, discriminant validity was assessed by checking that the latent variables’ average variance extracted was larger than the squared correlations between them (Mehmetoglu and Jakobsen, 2017, p. 305). Robust regression was used to investigate the relationship between TPB-factors and groups on the intention to use respirators. Robust regression was used due to issues with heteroskedasticity (Mehmetoglu and Jakobsen, 2017, pp. 334–338). Due to non-normally distributed factors, non-parametric tests were run to investigate differences between groups before and after intervention and differences within groups. Kruskal–Wallis and Mann–Whitney tests were used to investigate differences between groups at T0 and T1. Paired Wilcoxon rank-sum tests were applied to investigate changes before and after intervention for the different groups. Participation in the intervention groups or the control group was dummy coded such that intervention groups could be distinguished from the other intervention group and the control group. All analyses were conducted in STATA 15.0 (64-bit) for Windows.

### Ethics

The Regional Committee for Medical and Health Research Ethics declared that the project did not fall under the Norwegian health research legislation. In addition, the Norwegian Center for Research Data approved the method for collecting and storing data.

## Results

### Demographics

A total of 240 participants completed the post-intervention questionnaire. Reported demographics were based on those who had responded to both baseline and T1 (164/240). There were 146 males and 18 females, average age of 45.12 (*SD* = 13.05, range 18–69) and 75.15% had high-school or vocational diplomas. The distribution of demographics between groups over time were not significantly different. The distribution of participants from plants did not significantly differ over time.

### Confirmatory Factor Analysis

The model achieved the following fit statistics; a comparative fit-index (CFI) of 0.98 and a Root mean squared error of approximation (RMSEA) of 0.06. The *Organization* factor did not achieve satisfactory levels of discriminant and convergent validity, with average variance extracted of 0.45. Furthermore, the *Inconvenience* factor achieved a Raykov’s factor reliability coefficient of 0.66. Therefore, items within these factors could not be considered to measure the same underlying structure with confidence nor were they sufficiently different from each other. Even though some of these factors have issues, the researchers argue that the items themselves are of interest and have a subjective value. Therefore, they were not altered. See Supplementary Appendix Table A1 for statistics of the confirmatory factor analysis.

### Effects of Intervention

Kruskal–Wallis tests were performed to check for selection bias between those who answered only the baseline questionnaire and those who responded to both baseline and follow-up on all measured variables. No significant differences between groups were found.

Sum-scores of factors for the three groups were analyzed before and after interventions for comparison (Table 3). Both intervention groups (Group 1 and 2) showed an increase in *knowledge* regarding RPE use while the control group (Group 0) did not change. The median *attitude* score increased after intervention for both intervention groups, and was significantly higher in Group 2 compared to Group 1 and control after intervention. The *subjective norms* surrounding the use of RPE increased for group 2. Scores for *Perceived behavioral control* did not change for any group over time or between groups. Median scores for *organizational* items increased for Group 1. Both intervention groups showed a significant decrease in how much inconvenience they perceived by using RPE. Two research questions were central to this study, intent to use and rate of respirator use. I*ntention to use RPEs* as well as *rate of respirator use* did not change over the intervention, nor were there differences between groups in this analysis.

**TABLE 3:** Median and Mean scores on measured factors for the three intervention groups before and after intervention.

Factors		Time intervals		
		
	Group	Before	After	*p*
**Knowledge**				
Median (quartiles)	0	5.50 (4.50–6.00)	5.50 (5.00–6.25)	0.59
	1	5.00 (4.25–6.00)	6.00 (4.75–6.00)	<0.*01*
	2	5.50 (5.00–6.00)*	6.25 (5.75–6.75)*	<0.*01*
**Attitudes**				
Median (quartiles)	0	4.71 (3.43–5.71)	4.57 (3.86–5.71)	0.88
	1	4.29 (2.86–5.14)	4.43 (3.57–5.14)	<0.*05*
	2	4.43 (3.29–5.86)	5.29 (4.00–5.86)*	0.26
**Subjective norms**				
Median (quartiles)	0	4.33 (2.33–6.00)	4.33 (2.67-6)	0.22
	1	4.00 (2.00–5.33)	4.00 (2.33-5.33)	0.40
	2	3.50 (2.33–5.50)	4.33 (2.67–6.00)	<0.*05*
**Perceived behavioral control**				
Median (quartiles)	0	6 (2.5–6.5)	5 (1.5–6.5)	0.13
	1	4.5 (2.25–6.25)	5 (3.5–6.5)	0.15
	2	4.5 (3.5–6.5)	5.5 (2.5–6.5)	0.10
**Intention**				
Median (quartiles)	0	5.75 (4.5–7)	5.5 (4.75–6.75)	0.92
	1	5.75 (4.25–6.5)	5.75 (5–6.25)	0.15
	2	5.75 (5-6.75)	6 (5.00–6.75)	0.26
**Organization**				
Median (quartiles)	0	5.33 (4.5–6.33)	5.33 (4.50–6.17)	0.56
	1	5.50 (5–6.17)	5.67 (5.17–6.33)	<0.*05*
	2	6.00 (4.92–6.5)	**5.83 (5.42–6.5)**	0.28
**Inconveniences**				
Mean (*SD*)	0	3.35 (1.08)	3.43 (1.08)	0.85
	1	3.56 (1.10)	3.3 (1.15)	<0.*05*
	2	3.42 (1.02)	**3.10 (1.10)**	<0.*05*
**Rate of respirator use**				
Median (quartiles)	0	5.8 (4.7–6.8)	5.6 (5.2–6.6)	0.73
	1	5.8 (4.9–6.6)	5.8 (4.8–6.4)	0.78
	2	5.6 (4.6–6.4)	5.8 (5.00–6.60)	0.51

Including test statistics. Group 0 = control, Group 1 = Respirator Fit-testing, Group 2 = Respirator Fit-resting + Seminar “Dust and Health” note: **Bold,** Different from control. *Italics*, *different from before.* *, different from other intervention.

A multiple linear regression was calculated to predict Intention based on the independent variables; Attitudes, Subjective norms, Perceived control, Rate of respirator use, Group 1 and Group 2. A significant regression equation was found [*F*(6,228) = 38.75, *p* < 0.01], with an *R*^2^ of 0.55. Participants’ predicted Intention is equal to 1.53 + 0.28 (Attitudes) + 0.10 (Subjective norms) + −0.08 (Perceived control) + 0.30 (Group1) + 0.25 (Group 2), where Group 1 and Group 2 were coded as 0 = not participating and 1 = Participating. Participants Intention increased by 0.30 for participation in Group1 and 0.25 for participation in Group 2. Group 1 was the significant predictor of Intention. See Table 4 for details.

**TABLE 4:** Robust regression, after intervention.

**Intention**	***B***	**S(E)**	***t***	***p***	**β**	***n***	***F*(6,228)**	**P > f**	***R*^2^**	**Adj. *R*^2^**	**Root MSE**
						235	38.75	0.00	0.55	0.54	0.86

Attitudes	0.28	0.06	4.96	0.00	0.30						
Subjective norms	0.10	0.04	2.35	0.02	0.14						
Perceived control	–0.08	0.03	–3.00	0.00	–0.13						
Rate of respirator use	0.44	0.06	7.31	0.00	0.47						
Group 1	0.30	0.14	2.11	0.04	0.10						
Group 2	0.25	0.13	1.83	0.07	0.09						
Intercept	1.53	0.39	3.89	0.00							

*Differences between the control (Group 0) and intervention groups (Group 1 and 2) found in intention to use respirators, accounting for attitudes, subjective norms, perceived control and rate of respirator use.*

Results showed that 54% of the variance in intentions to use RPE was explained by this model. Participation in the intervention (Group 1) significantly increased the intention to use RPE (Group 2 showed a marginally significant increase) over and above the effects of core TPB variables (attitude, subjective norms, perceived behavioral control, and previous behavior).

## Discussion

The present study investigated the efficacy of a knowledge-based intervention containing two treatments aimed to increase workers’ knowledge and their general attitudes toward respiratory protective equipment (RPE) and their use. The intervention format was in part chosen because the authors wanted to test how fit-testing as an intervention would compare against a more common lecture-based intervention. As described earlier, Luong Thanh et al. (2016) showed small improvements in RPE use due to, among others, lecture based interventions. In addition, lecture based interventions are relatively easy to construct and perform. A review of the literature yielded no other studies addressing interventions among Norwegian smelter workers. Data was collected before and after intervention at six Norwegian smelting plants.

The design and content of the current intervention was partially based on research by Howie (2005), who provided a list of how to implement a successful program for RPE use. Howie pointed out the necessity of employee grounding in order to ensure the proper motivation for wearing RPE, including information of the consequences for the workers’ health from exposure, the necessity of having correct equipment available, knowledge of correct use and the importance of optimal fit. Graveling et al. (2011) also provided recommendations for proper RPE programs. They stated that all management levels need to understand the importance of, and need for RPE. These managers must also see to that appropriate RPE is supplied in sufficient quantities and that suitable provisions exist in order to clean, store and maintain RPE. Furthermore, they need to ensure that proper training and information is disseminated in the workforce. The RPEs need to fit individual employees, they should be comfortable and compatible with other PPE. The current study attempted to design an intervention intended to be used as a management tool to improve respirator use on employees using the aforementioned principles for RPE programs.

The current study aimed to deliver an efficient and applicable intervention to the smelting industry to optimize RPE use among the workers. The lecture was considered possible for non-scientific staff to implement for later use and course material was made accessible to all the smelters after the project, fit-testing should be performed by trained personnel.

Intervention groups (Group 1 and 2) were invited to RPE fit-testing, aiming to help the participant find a respirator that provided sufficient protection.

A comparable type of interactive training had been attempted in order to influence health workers’ attitudes toward RPEs. Carrico et al. (2007) performed an intervention consisting of classroom training combined with a visual bio-simulation training in order to increase RPE use for nurses working in a hospital. A control group received classroom training only. These authors did not report statistically significant difference between the two groups in respect to appropriate use of RPE for the nurses. However, they did find that nurses in the intervention group more often put a respirator on the patient compared to their control counterparts, suggesting that the intervention did have an effect. In the current study, no increase in rate of respirator use or intention to use respirators was observed. However, an increase in *knowledge*, *attitudes* toward RPE, *subjective norms* and a more positive view on the *organization* was found in addition to a decrease in the perception of inconvenience associated with RPE. There were no indications that conducting an intervention aiming to increase knowledge and attitudes toward the use of respirators affect smelting workers negatively.

Participants assigned to the control group did not report changes in any of the variables post intervention. Group 2 received the same RPE fit-testing procedure as group 1, in addition to a 45 min long educational lecture on dust, gas and fumes, health effects and protection. It was expected that the more comprehensive intervention given to group 2 would have a stronger effect. Both intervention groups showed increased scores on the *Attitudes* factor, although not statistically significant for group 2. Chatzisarantis and Hagger (2005) found that persuasive communications increased attitudes and intentions, but not reported behavior. However, there were some inconsistencies in the results of the current study. Group 1 increased their scores significantly on the *organization* factor, while group 2 did not, and vice versa for *subjective norms*. This indicated that going through RPE fit-testing resulted in a more positive view on the way the organization handled and administered RPE related issues. Since this effect was not observed in Group 2 it is possible that the content in the lecture may have modified the effect from the RPE fit-testing. It may also be a random effect. The increase on *subjective norms* in Group 2 may have indicated that participation in both fit-testing and the course increased perception of how colleagues evaluated RPE use. Accordingly, the content of the lecture may have altered perception of how people around them expect them to act. However, despite the described inconsistencies, the overall changes detected in the intervention groups compared to the control group suggest that the intervention was effective.

The clearest findings were increased *knowledge* scores for intervention groups. *Knowledge* increased for both intervention groups, and there were significantly higher scores in Group 2, as expected. Participants reported to know more about respirators, what they protected against and how they worked, after the intervention. The fact that the control group did not significantly change in knowledge score, indicated that the intervention measures were effective. Indeed, knowledge is considered an important precursor of behavior (Glanz and Bishop, 2010), although the causal relationship might not be a direct one. For instance, Fisher et al. (1996, p. 400) note that “*knowledgeable individuals are not necessarily motivated to change their behavior, and motivated individuals are not necessarily well informed*.” Donham et al. (2011) employed an intervention system (Certified Safe Farm) over a five-year period to e.g., increase the use of RPE, the knowledge of which type of RPE to use, understanding symptoms and possible health effects of exposure. These authors concluded that post intervention, farmers in the intervention group used RPEs more often than those in the control group, in addition, they suffered less acute symptoms of organic dust toxic syndrome. Dressel et al. (2007) reported similar results when measuring symptoms of asthma before and after educational interventions performed on farmers.

Perhaps the two most important outcome measures were participants’ *intention* to use RPE and *rate of respirator use*. Several studies have concluded that the use of respiratory protective equipment is not optimal in construction, manufacturing, health care, nuclear industry, farming and hazardous waste management (Salazar et al., 2001; Carpenter et al., 2002; Bryce et al., 2008; MacFarlane et al., 2008; Mitchell and Schenker, 2008; Tam and Fung, 2008; Han and Kang, 2009; Guseva Canu et al., 2013). Our own results indicated that most respondents in the current study had a high level of intent to use respirators at baseline and that they used respirators most of the time spent in exposed areas. The scores on *Intention* and *RPE use* did not change significantly post intervention, although regression analyses showed that allocation to an intervention group would predict an increased intention to use RPE. Given an average response rate of 34%, selection bias may have influenced the high baseline scores. Individuals who were more aware of health- and safety issues might have been more likely to participate. Non-responder analysis was not performed in this study, ergo this assumption will remain unanswered. Furthermore, the items included in the *Intention* factor were generated for the purpose of the current study and had not been validated elsewhere. It is possible that an optimized and more accurate instrument to measure *intention* would have been able to detect more subtle changes with greater accuracy. Nevertheless, the results are in line with existing literature reporting small increases in attitudes, knowledge and behavior after conducting similar interventions (Luong Thanh et al., 2016).

An observation often discussed at the plants with employees and management was that the observed non-compliance of respirator use was mostly a practical issue. In fact, previous analyses showed that only 4% of the respondents reported “laziness” or “bad excuses” as explanations to why they did not always wear RPE in exposed situations (Hegseth et al., 2018). However, comfort issues were reported as major reasons not to use RPE. Training in correct donning and finding a properly fitted respirator was expected to reduce discomfort and accommodate practical difficulties associated with RPE use. Indeed, the results showed that both intervention groups perceived significantly fewer practical challenges (*Inconveniences*) related to RPE use after the intervention compared to the control group. Improved knowledge of RPE and the possible health effects caused by exposure might have resulted in increased awareness of benefits of RPE use, which may have moderated the perception of practical disadvantages related to RPE use. However, this effect may be small, as indicated by previous findings, suggesting that beliefs about health benefits resulting from RPE influence use *less* than discomfort and inconvenience (White et al., 1988). The interventions may have provided participants with knowledge that they subsequently used to justify that RPEs are not as impractical or uncomfortable as previously experienced. It might therefore be that they do not experience less perceived inconveniences, but the knowledge they gained justified changes resulting in the measured decrease.

As reported from a study in a swine barn environment, the use of respirators can reduce negative acute health-effects in subjects not previously exposed to such environments (Dosman et al., 2000). There is no reason to assume that respirators would not help prevent non-acute issues as well. However, non-acute health issues that develop slowly over time are often considered less threatening than immediate and direct effects. Motivation to use RPE in order to prevent slowly progressing diseases such as COPD may therefore be moderate.

While implementation of interventions with an increase in awareness, attitudes, use of protective equipment and proper use of protective equipment are well documented in previous literature, proper randomization, use of controls and large samples are sometimes not well described or performed (Becker and Morawetz, 2004; Kim et al., 2012; Fu et al., 2013; Casalino et al., 2015; Navidian et al., 2015). Indeed, performing experimental procedures in applied settings where production is prioritized, can be impractical. Nevertheless, use of controls and random allocation into groups should be possible. Perry and Layde (2003) demonstrated that educational intervention sessions had an effect on participants’ knowledge, attitudes and use of protective equipment. Moreover, they noted that future interventions studies should ideally involve more than single-interventions.

Knowledge based interventions based on TPB have previously proven effective in increasing intentions and behavior, however no TPB-based intervention studies were found implemented in the Norwegian smelter industry. In the present study, one group received a single intervention, while the second group received two. Results did not show that either intervention were substantially superior to the other. Despite methodological short-comings, there seems to be a trend toward increased knowledge, attitudes and perceived inconveniences after the intervention. Future studies should investigate how comfort issues affect the use of RPE.

## Conclusion

The intervention, consisting of a lecture and/or respirator fit-testing, significantly improved the participants’ knowledge of RPE, attitudes toward RPE use, perceptions of their organization and the perception of how their surroundings considered their RPE use. Additionally, less hassle and practical issues were associated with RPE use after the intervention. Whereas mean comparisons between prior and post-intervention did not show changes in *intention to use RPE* or *rate of RPE use* over the intervention, regression analysis of post-intervention data revealed significantly greater *intention to use RPE* in each intervention group. Our findings indicate that knowledge-based interventions are useful to optimize workers’ motivations and attitudes toward RPE use in terms of increased knowledge and customization of the equipment, but that innate practical issues with RPE use are major reasons for non-compliance with regulations and have to be addressed through other measures.

### Practical Applications

The study intervention can be used as a framework for future health and safety work in industries where respiratory exposure represents a potential hazard. The current intervention was tailored for specific Norwegian smelting plants. The education package was based on exposure data from Norwegian smelters and addressed specific exposure scenarios for different plants, work-tasks and areas. To use the course in other industries, adjustments would be warranted to include specific exposure scenarios relevant to the designated work place. However, for most particle, gas and dust exposures, respiratory risks will be comparable, and the content of the course addressing this topic is employable in environments similar to smelters. The objective respirator fit-testing is applicable for all scenarios where employees are required to use respiratory protective equipment.

### Limitations

The data used in the study was founded on self-reports of attitudes, perceived coworker behavior, intention and rate of RPE use. Biases such as social desirability must always be taken into account when assessing data based on self-reports. Furthermore, the self-reports were cross-sectional in time. Meaning that there might be a difference in reported intention and actual intention in the specific situation of interest. The randomization method applied in this study could have influenced measures in that the three groups were present at all plants, meaning that the participants in reality were free to converse and share information out of the control of the researchers.

## Data Availability Statement

The datasets generated for this study are available on request to the corresponding author.

## Ethics Statement

Ethical review and approval was not required for the study on human participants in accordance with the local legislation and institutional requirements. The patients/participants provided their written informed consent to participate in this study.

## Author Contributions

ØR: design, data collection, analysis, and writing. MH and SF: design, data collection, editing, and writing. FS: editing and writing. ME: editing and writing. HV: design, data collection, editing, and writing.

## Conflict of Interest

We wish to draw the attention to the following facts which may be considered as potential conflicts of interest and to significant financial contributions to this work. Funding for this research was received from The Research Council of Norway (NOK 12 million, grant number: 245216) and The Norwegian Ferroalloy Producers Research Association and the *SiC* producers Washington Mills and Saint Gobain (NOK 6 million). SF worked for Elkem from August 2013 to August 2014 as Corporate advisor in occupational hygiene and toxicology. However, SF was not working for Elkem during the project. The remaining authors declare that the research was conducted in the absence of any commercial or financial relationships that could be construed as a potential conflict of interest.
