# Supplementary material for: The Effect of a Knowledge-Based Intervention on the Use of Respirators in the Norwegian Smelter Industry
Source: Front Psychol. 2020 Feb 20;11:270. doi: 10.3389/fpsyg.2020.00270 (PMC7044339; doi:10.3389/fpsyg.2020.00270)
Supplement: Supplementary file 1 [file Data_Sheet_1.docx]

**Appendix**

**Table A1.** *Confirmatory factor analysis of subjective measures.*

| Constructs and indicators | Factor Loadings | Raykov's composite reliability | Variance Extracted | Mean | Std. Dev. |
| --- | --- | --- | --- | --- | --- |
| ***Knowledge*** |  | .84 | .56 |  |  |
| I am confident that the respirator works as intended. | .60 |  |  | 4.74 | 1.63 |
| I feel I have been provided good information why I should wear a respirator. | .94 |  |  | 6.45 | 1.21 |
| I know what the respirator protects against. | .67 |  |  | 4.10 | 1.70 |
| ***Organization*** |  | .70 | .44 |  |  |
| The organization is willing to provide personalized protective equipment. | .54 |  |  | 5.64 | 1.83 |
| The company focuses on the use of respirators. | .78 |  |  | 4.04 | 2.16 |
| Management seems focused on improving the working climate. | .62 |  |  | 5.83 | 1.76 |
| Employee suggestions on improvements are taken into consideration properly and discussed openly. | .68 |  |  | 3.62 | 2.24 |
| ***Inconveniences*** |  | .66 | .50 |  |  |
| It is impossible to follow respirator guidelines during some work-tasks. | .66 |  |  | 4.53 | 2.44 |
| It’s practically impossible to follow regulations regarding respirator use. | .70 |  |  | 5.67 | 1.55 |
| Sometimes I don’t bother changing respirator even though I know I should. | .56 |  |  | 4.46 | 2.42 |
| ***Rate of respirator use*** |  | .83 | .63 |  |  |
| ***“During the last work-week, I have ...”*** |  |  |  |  |  |
| Always used the respirator in exposed areas | .75 |  |  | 2.28 | 2.01 |
| Always used the respirator in appropriate situations | .90 |  |  | 5.27 | 1.89 |
| Always used respirators according to regulations. | .72 |  |  | 5.68 | 1.68 |

**Table A2:** *List of item wording for Attitudes, subjective norms, Perceived control and Behavioral intention.*

| Constructs and indicators |
| --- |
| ***Attitudes*** |
| Regularly using a respirator during the next work-week would be ... |
| a … Very harmful - Not harmful at all. |
| b … Very uncomfortable - Very comfortable. |
| c … Very impractical - Very practical. |
| d … Very cumbersome - Not cumbersome at all. |
| e … Not desirable at all - Very desirable. |
| f … Very annoying - Not annoying at all. |
| g … Hindering - Not hindering at all. |
| h … Very exhausting - Very easy. |
| ***Subjective norms*** |
| Most workers like me always use a respirator while working. |
| My colleagues always wear respirators during the work-week. |
| My colleagues at the plant usually wear a respirator at work. |
| ***Perceived control*** |
| It's up to me whether or not I use the respirator during a work-week. |
| I am in charge of which situations I use the respirator in during a work-week. |
| ***Intention*** |
| I'm going to use the respirator even though it makes it harder to breathe. |
| I am going to use the respirator in all required situations next work-week. |
| I am going to use the respirator even though it is warm and uncomfortable. |
| I am going to use the respirator even though it is impractical. |
